# Prevalence of Painful Temporomandibular Disorder Symptoms Among Professional and Student Musicians: An Online Survey

**DOI:** 10.1111/joor.13868

**Published:** 2024-09-30

**Authors:** Suzanne Z'Graggen, Dominik A. Ettlin, Elena Alessandri, Werner J. Z'Graggen, Martin Schimmel

**Affiliations:** ^1^ School of Music, Lucerne University of Applied Sciences and Arts Lucerne Switzerland; ^2^ Department of Restorative, Preventive and Pediatric Dentistry School of Dental Medicine, University of Bern Bern Switzerland; ^3^ Department of Reconstructive Dentistry and Gerodontology School of Dental Medicine, University of Bern Bern Switzerland; ^4^ Department of Neurosurgery Inselspital, University Hospital Bern, University of Bern Bern Switzerland; ^5^ Department of Neurology Inselspital, University Hospital Bern, University of Bern Bern Switzerland; ^6^ Division of Gerodontology and Removable Prosthodontics University Clinics of Dental Medicine, University of Geneva Geneva Switzerland

**Keywords:** music, music instruments, musical performance, musicians, survey, temporomandibular disorders, TMD pain screener

## Abstract

**Background:**

Within the fields of medicine and music, increasing attention is given to evidence indicating music performance being an occupational risk factor for temporo‐mandibular disorders (TMD).

**Objectives:**

Assessment of self‐reported painful TMD symptoms among student and professional musicians.

**Methods:**

Using Survey Monkey software, the German version of the ‘TMD‐Pain‐Screener’ was distributed electronically to professional and student musicians in Europe. Supplementary questions addressed age, gender, daily playtime, instrument type and type of professional practice. Results are presented as median [interquartile range].

**Results:**

The TMD‐pain‐screener was completed by 492 participants. Among them, 96 (19.5%) reported painful TMD (Pain_pos) and 396 did not experience pain (80.5%, Pain_neg). Pain_pos participants were significantly younger (Pain_pos: 34 years [26; 46], Pain_neg: 44 years [30; 56], *p* = 0.0003), had less work experience (Pain_pos: 15.5 years [6; 25], Pain_neg: 20 years [10; 32], *p* = 0.009), had fewer performances/year (Pain_pos: 20/year [10; 45], Pain_neg: 30/year [12; 53.5], *p* = 0.03) and were predominantly female (OR = 3.22 [1.87, 5.74], *p* < 0.0001). Comparisons among music performance types revealed no statistical significance in the overall test (*p* = 0.13), although ‘keyboard’ (OR = 2.99 [0.58, 30.12]), ‘upper string’ (OR = 2.31 [0.43, 23.63]) and ‘singer’ (OR = 2.14 [0.44, 20.75]) stood out compared to the reference group ‘lower string’ (OR 1.00). Organ players formed the largest group and showed a comparatively low prevalence of Pain_pos (16%), compared to other keyboard instruments (Pain_pos 30.2%).

**Conclusions:**

Prevalence of TMD pain was highest among young inexperienced female musicians. Playing keyboard instruments (other than organ) or upper strings instruments were frequently associated with painful TMD screening. An improved understanding of causes, implementation of preventive measures, professional guidance and a biopsychosocial health care perspective may decrease this occupational risk while maintaining the health benefits of music.

## Graphical abstract

Odds Ratios (OR) for TMD status (yes/no) according the main instrument groups.

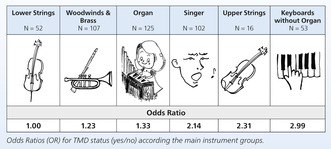

## 1 Introduction

Music has been associated with health benefits [1, 2]. A substantial portion of the population engages in musical activities. For example, approximately 20% of Swiss individuals play an instrument or sing in a choir, and in Germany, about 9 million people aged 14 years or older play an instrument [3, 4].

Musicians, many of them with a dedicated strive for excellence, may experience that the pursuit of perfection leads to significant challenges. Among these, playing‐related musculoskeletal disorders (PRMDs) stand out as a significant concern that affects numerous musicians across different genres and skill levels [5]. The repetitive, precise and particular movements associated with playing musical instruments or singing, combined with hours of practice and performance, can lead to discomfort in corresponding body parts, including the orofacial region [6, 7]. Understanding and addressing PRMDs in professional musicians is crucial for preserving their well‐being and sustaining their level of performance.

The orofacial system engages in activities like chewing, speaking and notably in musicians, playing instruments or singing [8, 9]. It plays a pivotal role in executing various musical techniques, such as embouchure control in wind instruments or shifting and plucking in string instruments, thereby exposing tissues to unique biomechanical stressors [10].

These movements essential for making musicians can lead to temporomandibular disorders (TMD), a collective term describing a range of conditions affecting the temporomandibular joints (TMJ) and surrounding muscles, ligaments, nerves and other tissues. Characteristic symptoms include orofacial pain, headaches, neck and shoulder pain, limitations in mouth opening and jaw joint noises. TMD encompasses not only bodily impediments but also may further involve psychological and social burdens [11, 12].

The established evidence‐based gold standard for screening and diagnosis are the Diagnostic Criteria for Temporomandibular Disorders DC/TMD questionnaires and examinations, which comprise an ‘axis I’ reflecting the pain and physical diagnosis and an ‘axis II’ representing the psychosocial status. Positive screening tests may be verified clinically with confirmatory tests [13]. The ‘TMD Pain Screener’ questionnaire is the recommended pain‐screening test of the DC/TMD axis I and can be used to screen for self‐reported TMD symptoms, both on an individual as well as on an epidemiological level [14]. The longer version consists of six items related addressing jaw pain, mandibular motion, habits and orofacial function [15]. Its diagnostic accuracy varies depending on the study population with sensitivities reported between 0.85 and 0.99 and specificities between 0.52 and 0.97 [14, 16].

In a meta‐analysis of studies utilising the Research Diagnostic Criteria for Temporomandibular Disorders (RDC/TMD) or DC/TMD test batteries, a 54% TMD prevalence was observed in musicians [17]. These data indicate that TMDs are a relevant condition within this professional group, but may differ between different types of musical instrument, or other forms of music performance such as singing and conducting music [18].

The intricate relationship between playing musical instruments or singing and the development of TMD has drawn increasing attention within the medical and musical communities [19, 20, 21].

Understanding the prevalence, risk factors, development and implications of TMD in musicians is crucial for early detection and effective management of these disorders. ‘Therefore, a study was designed as an online survey to gather data from music students and professional musicians on their experiences with orofacial pain, demographic details and music practice habits. The survey had two parts: the first collected general information such as age, gender, and musical background, while the second part used the TMD Pain Screener to screen for jaw‐related pain and dysfunction. The survey was distributed through email and social media to a targeted group of classical, jazz, and folk musicians, with responses categorized based on musical performances for analysis’. The null hypothesis was ‘There is no difference in the prevalence of self‐reported painful TMD between the different types of music making and the level of professional experience’.

## 2 Materials and Methods

This study was evaluated by the Cantonal Ethics Committee of Bern and was deemed to not fall under the Swiss Federal Human Research Act (KEK Req‐2022‐01315).

An online survey with the Survey Monkey software (https://www.surveymonkey.com, Momentive Global Inc., San Mateo, CA, USA) was set‐up. The survey consisted of two parts. In the first part, the participants were asked to provide general information about age, gender, main instrument, work experience, daily playtime of the instrument/singing, number of performances per year, type of professional music practice (student or professional musician) and level of music education.

The second part comprised the German translation of the longer version of the TMD Pain Screener [15]. It contains six self‐reported items related to jaw pain, mandibular motion, habits and orofacial function with a score range between 0 and 7. According to previous publications, a score ≥ 3 was considered a positive screening result for painful TMD (Pain_pos). Those with < 3 points show a negative screening test result (Pain_neg) [15].

Between 12 April 2023 and 21 June 2023, the link to the online survey was sent together with a short description of its aim and scope directly to student and professional musicians via email. Further inclusion criteria comprised being older than 18 years without upper age limit, and being capable of understanding the German language. There were no restrictions applied according to the music genres, such as classical music, jazz, rock, folk, etc. Because of the personal background of the main investigator (SZ) and the institutional alignment of the School of Music, Lucerne University of Applied Sciences and Arts, Lucerne (HSLU‐M), predominantly classical, jazz and folk musicians received the questionnaire. A total of 201 emails were sent by SZ to her professional network (the German, Swiss and Austrian professional associations of music, church music, orchestra, educational music schools, choirs, chamber music ensembles and freelance musicians). Certain multipliers such as heads of music schools, were encouraged to forward the link to their networks. Additionally, 841 emails were distributed among the students and staff of the HSLU‐M. Furthermore, the study was advertised on social media platforms such as LinkedIn, Facebook and Instagram.

The participants were classified according to instrument‐specific playing styles/singers. This classification was chosen because it refers to the playing‐related demands of specific anatomical parts of the body (i.e., Low Bow, High Bow, Singer, Organ, Keyboards [without Organ], Wind & Brass). Instrument groups played by fewer than 10 participants were excluded from further analysis. The construction of the instruments was not the subject of the study; it must be assumed that both historical and modern instruments were played by the participants.

### 2.1 Statistical Analysis

Data were exported from Survey Monkey into Microsoft Excel and participants with missing data on the TMD Pain Screener were omitted.

Statistical analysis was performed with the free statistical software R (version 4.1.0) by a professional statistician [22]. Throughout, *p* ≤ 0.05 were considered statistically significant.

Descriptive statistics in the form of medians (25%–75% quantiles for continuous variables, IQR) and frequencies (percentages) were used to summarise variables of interest. Participants were grouped according to their TMD Pain Screener results into TMD‐groups (Pain_pos/Pain_neg). Variables of interest were compared between TMD‐groups using Mann–Whitney tests. Continuous and exact Fisher tests (multivariate extension) were used for categorical variables of interest. For the latter, odds ratios (ORs) were calculated.

All significant variables from TMD‐group‐comparisons except for the variable age (assumed to strongly correlate with variable Work Experience) were then put into one large multivariate logistic regression model. A backward elimination procedure minimising the Akaike Information Criterion (AIC) was used to reduce the model to only risk factors, including ORs and 95% confidence intervals (CIs). The Goodness‐of‐fit of the final model was assessed with the help of the Hosmer–Lemeshow test.

## 3 Results

Five‐hundred and seventy‐three (*n* = 573) responses were received. The TMD‐Pain‐Screener (second part) was fully completed by 492 participants. Five musicians did not provide general information in the first part but were included in the analysis of the second part. An exact response rate could not be determined, as the link to the survey was also forwarded to an unknown number of student or professional musicians by musical directors, for example, orchestra conductors and music school directors.

Four groups of music performance types were omitted from further analysis because of the small sample size *n* ≤ 10 in each group: conducting, guitar, percussion instruments, as well as ‘music and movement’. The groups that qualified for statistical analysis were lower string instruments (double bass, violoncello and viola da gamba), upper string instruments (violin and viola), singers (soprano, mezzo‐soprano, alto, tenor, baritone and bass), organ, keyboard instruments (piano, harpsichord and accordion) and wind and brass instruments (saxophone, clarinet, flute, recorder, hautboy, trumpet, trombone, French horn and tuba).

Seventy‐nine per cent (79.5%) of the participants studied or worked in Switzerland. General information on age, gender and country of origin is provided in Table 1. Further information related to level of musical education, along with work experience, daily playing time and number of performances is shown in Table 2.

**TABLE 1:** General information on the study participants who provided information in the first part of the survey.

Variables	Students median (IQR) *N* = 95	Professional musicians median (IQR) *N* = 392	Total with known profession median (IQR) *N* = 487
Age (years)	23 (21–26)	47 (35–56)	43 (29–55)
Gender			
Male	*N* = 28 (13.4%)	*N* = 181 (86.6%)	*N* = 209 (100%)
Female	*N* = 66 (24.2%)	*N* = 207 (75.8%)	*N* = 273 (100%)
Diverse	*N* = 0 (0%)	*N* = 2 (100%)	*N* = 2 (100%)
Country			
Switzerland	*N* = 82 (21.2%)	*N* = 305 (78.8%)	*N* = 387 (100%)
Austria	*N* = 1 (3.7%)	*N* = 26 (96.3%)	*N* = 27 (100%)
Germany	*N* = 9 (24.3%)	*N* = 28 (75.7%)	*N* = 37 (100%)
Other	*N* = 1 (7.7%)	*N* = 12 (92.3%)	*N* = 13 (100%)

Abbreviation: IQR, interquartile range.

**TABLE 2:** Playing‐related parameters of all participants who provided information in the first part of the survey.

Variables	Students median (IQR) *N* = 95	Professional musicians median (IQR) *N* = 392	Total with known profession median (IQR) *N* = 487
Work experience (year)	3 (1–6)	25 (13–33)	20 (8.5–30)
Practice time (h/day)	3 (2–4)	2 (2–4)	3 (2–4)
Number of performances (*n*/year)	20 (8–35.75)	30 (12–60)	30 (12–50)
Level of education			
Bachelor	*N* = 18 (29.5%)	*N* = 43 (70.5%)	*N* = 61 (100%)
Student	*N* = 65 (82.3%)	*N* = 14 (17.7%)	*N* = 79 (100%)
Master	*N* = 8 (5.1%)	*N* = 149 (94.9%)	*N* = 157 (100%)
Two or more masters	*N* = 3 (2%)	*N* = 146 (98%)	*N* = 149 (100%)
Degree ‘Church Music C’	*N* = 0 (0%)	*N* = 18 (100%)	*N* = 18 (100%)
Layman	*N* = 0 (0%)	*N* = 12 (100%)	*N* = 12 (100%)

Abbreviation: IQR, interquartile range.

Among the 492 participants who completed the TMD‐Pain‐Screener, 96 (19.5%) were Pain_pos. Pain_pos were significantly younger, had less work experience, had fewer performances/year and were predominantly female (Table 3). As for the groups of music performance, there was no statistical significance in the overall test (*p* = 0.13); however, ‘keyboard’ (OR = 2.99 [0.58, 30.12]), ‘upper string’ (OR = 2.31 [0.43, 23.63]) and ‘singer’ (OR = 2.14 [0.44, 20.75]) stood out compared to the reference group ‘lower string’ (OR 1.00) (Table 3).

**TABLE 3:** Participants grouped according to their TMD status (yes/no) who provided information in the second part of the survey (*n* = 492), descriptives and univariate analysis.

Variables	TMD = no median (IQR) *N* = 396	TMD = yes median (IQR) *N* = 96	*p*
Age (year)	44 (30–56)	34 (26–46)	*p* = 0.0003
Work experience (year)	20 (10–32)	15.5 (6–25)	*p* = 0.009
Practice time (h)	3 (2–4)	3 (2–4)	*p* = 0.44
Number of Performances/year	30 (12–53.5)	20 (10–45)	*p* = 0.03
Gender (year)			
Male	191 (90.1%)	21 (9.9%, reference)	*p* < 0.0001
Female	203 (73.8%)	72 (26.2%, OR = 3.22 [1.87, 5.74])	
Level of education			
Bachelor	49 (80.3%)	12 (19.7%, reference)	*p* = 0.03
Student	55 (68.8%)	25 (31.2%, OR = 1.85 [0.79, 4.49])	
Master	136 (85.5%)	23 (14.5%, OR = 0.69 [0.30, 1.65])	
Two or more Masters	119 (79.9%)	30 (20.1%, OR = 1.03 [0.47, 2.40])	
Main instrument groups			
Low bow	14 (87.5%)	2 (12.5%, reference)	*p* = 0.13
High bow	39 (75%)	13 (25%, OR = 2.31 [0.43, 23.63])	
Singer	78 (76.5%)	24 (23.5%, OR = 2.14 [0.44, 20.75])	
Organ	105 (84%)	20 (16%, OR = 1.33 [0.27, 12.97])	
Keyboards	37 (69.8%)	16 (30.2%, OR = 2.99 [0.58, 30.12])	
W & B wind	91 (85%)	16 (15%, OR = 1.23 [0.24, 12.17])	
Profession			
Student	68 (71.6%)	27 (28.4%, reference)	*p* = 0.02
Professional musician	323 (82.4%)	69 (17.6%, OR = 0.54 [0.31, 0.94])	

*Note:* Statistical tests: For continuous variables, Mann–Whitney test; group frequencies, exact Fisher test. For categorical values, odds ratios (ORs) including 95% CI for TMD status (yes/no) where the first mentioned group is the reference group.

The multivariate analysis with backward selection and respective risk factor assessment resulted in a model that consisted of the two main variables work experience and gender. With each year of work experience, the odds of having TMD decrease by 2% (OR = 0.98 [0.96, 1.00], *p* = 0.04). Compared with male participants, female musicians had 2.96 times higher odds of reporting a painful TMD (OR = 2.96 [1.64, 5.62], *p* = 0.0005).

## 4 Discussion

This online survey using the long version of the TMD‐Pain‐Screener questionnaire revealed that TMD pain is reported more frequently by music students than professional musicians, and those with female gender, younger age, fewer performance and less work experience than their respective controls. Although the overall statistical test showed no difference between the groups of music performance types, players of keyboard instruments, upper string instruments and singers were more than twice as likely to report pain compared to musicians playing lower string instruments, who screened positive in only 12.5% of the cases. Organ players and participants in the group playing wind and brass instruments showed only slightly higher ORs than the reference group playing lower string instruments.

### 4.1 Strengths and Weaknesses of the Study

This study was planned and executed as an interdisciplinary research project, involving professional musicians and specialists in orofacial pain and dysfunction. In this way, in‐depth details about performing music and individual participant‐based factors important to the target group could be investigated. Often, dental and medical research lacks this ‘patient‐centred’ approach which may lead to paternalistic decisions in healthcare, and subsequently poor acceptance and compliance for prophylactic and therapeutic measures [8, 23].

Furthermore, through the professional network of the main investigator (SZ), a large sample size among the target group could be reached in a short period of time. The use of an online platform facilitated the distribution even further. However, the free distribution of the link to the survey also led to a rather large variety among instrument groups and through the strong focus of the SZ network, there is an over‐representation of church musicians, that is, professional organ players. For the same reason, it should be noted that the musicians surveyed were mainly from the following musical styles: classical, jazz and folk music. Musicians from the fields of pop or rock music were unlikelier to be included.

It would have been preferable to include a control group of individuals not performing music, but this would have been quite challenging as the prevalence of playing an instrument or singing in a choir is quite high. Furthermore, a control group would have had to be matched at least in age and gender. Therefore, it was decided to compare the current prevalence figures with evidence from the literature.

Another shortcoming is the lack of a sample‐size calculation, and hence, the lack of power in some statistical tests involving instrument groups with small sample sizes. This might have led to non‐significant findings, like the absence of an overall significance when comparing the groups of music performance. It was therefore chosen to calculate OR for the subgroups to describe the statistical trends.

A further weakness of the chosen form of distribution is that it is not possible to follow‐up on the student participants after they have graduated and gained more experience. This might have been interesting as it may be inferred from the current results, that an increase in experience might lead to a decrease in reported orofacial pain which could be related to stress reduction through growth in terms of proficiency. Also, there could be an inclusion bias. Additionally, since this was a cross‐sectional rather than a longitudinal study, we lack insights if professional musicians already experienced TMD symptoms when they were students.

### 4.2 Strengths and Weaknesses in Relation to Other Studies, Discussing Particularly Any Differences in Results

Campos et al. [17] reported in their meta‐analysis an overall combined prevalence of TMD of 53.9%. This number is higher than that found in our study. It must be noted that in their systematic literature review, mostly studies using the RDC/TMD, or DC/TMD test batteries were included. There are only few studies using the TMD‐pain screener alone to detect orofacial pain in specific groups; for example, Chuinsiri and Jitprasertwong [12] reported a prevalence of 22.2% in their sample which is similar to the present results. However, it has to be noted that their study population comprised only of patients in a dental clinical setting, and of course, one of the main reasons to seek help from a dentist is pain [24].

Our study shows similar prevalence rates in singers, wind and brass instruments and upper string instruments than an earlier study [7]. However, PRMDs are frequently reported by musicians and some may be more likely related to myofascial pain and temporomandibular joint (TMJ) pain. The assumption that the primary cause of orofacial pain in musicians originates from the intense use of the orofacial system when playing an instrument or singing has been supported by a recent study that did not detect differences in psychological distress, pain coping and disability among music performance groups experiencing orofacial pain [19].

In this study, the pooled prevalence of orofacial pain in professional musicians confirms findings from a recent questionnaire‐based study in the Netherlands [7]. However, in this analysis, a high prevalence of self‐reported orofacial pain was present in keyboard players, which contradicts findings from van Selms et al. [7]. Pianoplayers in the Dutch study were even used as a negative control group, as it was assumed that other musculoskeletal structures other orofacial structures were used. In this study, 30.2% of musicians playing keyboard instruments experienced TMD pain. These discrepancies require further exploration in future studies. It can be speculated that the occurrence of TMD cannot only be attributed to the direct use of the orofacial system for playing an instrument and may have a wider pathophysiological origin. Accompanying orofacial movements can often be observed in keyboard players while performing and might explain to some extent the high frequency of TMD. Anatomically, the hand representation in the primary motor and sensory cortex is adjacent to the orofacial region [25]. Intensive piano practice has been shown to influence both the structure and excitability of the sensory‐motor representation of the hand and also of other brain areas [26]. Furthermore, maladaptive central nervous changes are a well‐known phenomenon in musicians leading to, for example, focal dystonia [27]. Hence, the occurrence of TMD might be facilitated to some extent by such central adaptive and maladaptive mechanisms.

In terms of playing style, the organ belongs to the keyboard instruments. However, it is puzzling that despite the high complexity of the instrumental requirements (such as playing with the hands on multiple manuals and simultaneously using the feet for the pedalboard), there was a lower prevalence of reported TMP‐related pain. To the best of our knowledge, this is the first study to report on prevalence rates of TMD pain in organ players (16%, compared to 30.2% in other keyboard instrumentalists). The enrolled participants were mostly professional church musicians, with a very high frequency of performances/year, and therefore, had profound experience and routine in their play, which might be one reason for the lower prevalence. Furthermore, the cortical representation of the foot in the primary motor and sensory cortex, which is medial to the hand area and therefore further away from the face area, which is itself lateral to the hand area, might be a factor (see above).

### 4.3 Stress and Anxiety

Stress and anxiety are very common among professional musicians, especially among music students [28]. Performances in front of professional colleagues (e.g., in an orchestra) and/or audiences can be a stressful situation for musicians, as their musical abilities are constantly subjected to critical evaluation. Under extreme circumstances, existential fears may arise because failure could have direct consequences on future engagements or the musician's reputation as an interpreter. As a result, professional musicians often struggle with fears of failure and a strong pursuit of perfection. These emotional states and attitudes may negatively impact on musicians' health, including orofacial pain [29, 30].

In this study, the significantly higher prevalence of orofacial pain among student musicians compared to more experienced musicians may partly be explained by less developed coping strategies among students. It has been stated in the literature that the frequency of performances may be positively correlated with the occurrence of TMD [31, 32]. Current research suggests that stress and anxiety might be stronger factors in developing performance‐related pain and disability than previously discussed [33]. In future clinical studies investigating orofacial pain in musicians, stress, fear of performance and anxiety should be evaluated as contributing factors.

### 4.4 Meaning of the Study: Possible Mechanisms and Implications for Clinicians or Policymakers

The implications of TMD pain in musicians go beyond physical discomfort; it can significantly impact their performance, leading to decreased endurance, diminished technical ability and even a loss of motivation to practice or perform. For professional musicians whose livelihoods depend on their ability to play at an exceptional level, this pain can be particularly challenging, affecting their careers and overall well‐being. This might be a reason why the prevalence of TMD pain was significantly higher in students (28.4%) than in professionals (17.6%). TMD pain could well be a reason for stopping playing music in students.

Addressing TMD pain in musicians requires a multifaceted approach. Firstly, raising awareness and providing education among musicians about proper posture, ergonomics and techniques that minimise strain on the jaw and facial muscles are essential [6].

Seeking professional guidance from healthcare practitioners specialised in orofacial pain, such as dentists, physical therapists or specialists in TMJ disorders, is crucial. These experts can provide personalised assessments, recommend specific exercises or therapies, and, if necessary, suggest interventions like the acquisition of jaw muscle relaxation skills, splints or mouthguards to alleviate pain and prevent further damage [34].

### 4.5 Unanswered Questions and Future Research

The prevalence of TMD among musicians is a subject of growing concern. Research indicates a higher incidence of TMD symptoms, including jaw pain, muscle fatigue and limited jaw mobility, among instrumentalists compared to the general population. Furthermore, the multifaceted nature of TMD manifestations, which can vary from myofascial pain to joint disorders like disc displacement or arthritis, underscores the complexity of diagnosing and managing these conditions in musicians.

Identifying and addressing TMD pain in its early stages, or even preventing it from developing in the first place, is paramount to mitigate its impact on musicians' performance and prevent potential long‐term complications. Effective screening protocols tailored for musicians can serve as a proactive measure in early detection. These screening methods encompass a comprehensive assessment of musicians' musculoskeletal health, jaw function and associated pain or discomfort. Specialised diagnostic tools, including questionnaires, clinical examinations, imaging techniques and jaw muscle activity assessment by ecological momentary monitoring all may offer valuable insights into the presence and severity of TMD‐related symptoms, aiding in timely intervention and management strategies.

## 5 Conclusion

In conclusion, TMD pain is prevalent among musicians, predominantly in female music students. To clarify the occupational burden, symptom variability during the course of time necessitates further investigation. An improved understanding of the causes, the implementation of preventive measures, professional guidance and a biopsychosocial healthcare perspective may decrease this occupational risk while maintaining the general health benefits of music.

## Author Contributions

**Suzanne Z'Graggen:** development of the research question, methodology, data acquisition, data interpretation and contribution to the manuscript; **Dominik A. Ettlin:** development of methodology, data interpretation and critical revision of the manuscript; **Elena Alessandri:** methodology and critical revision of the manuscript; **Werner J. Z'Graggen:** development of methodology, data interpretation and critical revision of the manuscript; **Martin Schimmel:** development of the research question, data analysis, ethics, funding and contribution to the manuscript.

## Conflicts of Interest

The authors declare no conflicts of interest.

### Peer Review

The peer review history for this article is available at https://www.webofscience.com/api/gateway/wos/peer‐review/10.1111/joor.13868.

## Data Availability

The original data of the study can be made available upon reasonable request.
